# Off-Label Use of Teriparatide in Spine

**DOI:** 10.7759/cureus.16522

**Published:** 2021-07-20

**Authors:** Panagiotis Drakopoulos, Dimitrios A Flevas, Ioannis P Galanopoulos, Panagiotis Lepetsos, Christos Zafeiris

**Affiliations:** 1 Laboratory for the Research of the Musculoskeletal System, University of Athens, KAT Hospital, Athens, GRC; 2 Orthopaedics, Thriasio General Hospital, Athens, GRC; 3 Arthroscopy and Orthopaedic Surgery, Metropolitan General Hospital, Athens, GRC; 4 Trauma and Orthopedics, KAT Hospital, Athens, GRC; 5 Orthopaedics and Spine Surgery, Metropolitan General Hospital, Athens, GRC

**Keywords:** off-label, teriparatide, spine, spinal fusion, parathyroid hormone

## Abstract

Teriparatide belongs to osteo-anabolic compounds and has been used in recent years to treat patients with osteoporosis, with the benefits of increased bone density. Its osteo-anabolic action has led to the investigation of the use of teriparatide for the improvement of bone quality. Apart from the enhancement of fracture union, teriparatide has been extensively studied in the promotion of fusion rate after spinal fusion. This study summarizes the preclinical and clinical results of the off-label use of teriparatide in the spine, and specifically its intermittent administration after instrumented spinal arthrodesis along with its impact on the spinal bone quality and spinal bone mineral density.

## Introduction and background

Parathyroid hormone (PTH) is a polypeptide consisting of 84 amino acids produced by parathyroids and involved in maintaining calcium and phosphate homeostasis. PTH is secreted as a response to the reduction in calcium serum levels and acts by increasing calcium release from bones and reabsorption from the renal tubules while increasing calcium absorption from the small intestine via the 1,25-VitD synthesis (VitD3). While PTH is included in osteoclasting agents, when it is administered periodically in small doses, it stimulates bone tissue production. The recombinant analogue of PTH (rPTH or PTH1-34) or teriparatide has been used since 2002 as an osteoanabolic agent with great success. It is the N-terminal portion of PTH, consisting of the first 34 amino acids. This polypeptide contains all the classical biological and biochemical effects of the entire PTH molecule, that with the 84 amino acids [PTH (1-84)]. Since PTH stimulates bone production, areas with an increased rate of bone metabolism, such as cancellous bones (e.g. lumbar spine), will exhibit the greatest increase in bone density. The microscopic structures of the skeleton are improved by increasing the thickness of cancellous bone, while cortical bone improves both its structure and its thickness [1].

Teriparatide belongs to osteo-anabolic compounds and has been used in recent years to treat patients with osteoporosis, and has the benefit of increasing bone density. Its osteo-anabolic action has led to the investigation of the use of teriparatide for the improvement of bone quality and restoration of other bone diseases (incomplete fracture of fractures, osteoarthritis, incomplete osteogenesis, dentistry, endocrine diseases, etc.). Teriparatide promotes the maturation of circulating osteoblast precursors and differentiation of lining osteoblasts, triggers the formation of new bone tissue by the preexisting osteoblasts, and limits osteoblast and osteocyte apoptosis [2]. Furthermore, it seems that it may have a potential role in fracture healing by enhancing various processes involved in the initial formation of endochondral bone and in the construction of the primary callus. To date, most studies show positive results, but as the literature consists almost entirely of case reports or case series, there is a clear risk of bias. 

The purpose of this paper is to summarize the relevant information from the international bibliography and to draw conclusions about the progress of science in the subject.

## Review

I. On-label use of teriparatide

Officially, teriparatide is indicated for the treatment of osteoporosis in postmenopausal women and in men at increased risk of fracture. It is also used to treat osteoporosis associated with prolonged and systemic glucocorticoid therapy in women and men. Finally, it is also used in patients who, while under treatment with bisphosphonates, suffer from osteoporotic fractures or fragility fractures or have a decreasing bone density [3,4]. Currently, based on the World Health Organization guidelines, the recommended dose of teriparatide is 20 micrograms once a day. The maximum total duration of treatment with teriparatide should be 24 months. This 24-month treatment should not be repeated over the life of the patient. Patients should receive calcium and vitamin D supplements if they have a low-nutrition diet. 

II. Off-label use of teriparatide

It seems that teriparatide may have a potential role in fracture healing by enhancing various processes involved in the initial formation of endochondral bone and in the construction of the primary callus. The healing process is similar to the process of fetal and infant bone growth and it appears that PTH specifically affects endochondral ossification by stimulating the proliferation and differentiation of osteoprogenitor cells and increasing the production of bone matrix proteins. During this process, periarticular chondrocytes differentiate into extensively proliferating chondrocytes which eventually cease their proliferation and differentiate into hypertrophic chondrocytes. Although most studies show positive results, there is a clear risk of bias since the literature consists almost entirely of case reports or case series. Most of the reports on the successful use of PTH include patients with fractures who either have healing problems or have some features suggesting that healing can be compromised. The sporadic use of teriparatide for the enhancement of fracture healing stems from the success of teriparatide in the treatment of osteoporosis [5].

In the literature, teriparatide has been used in the healing of atypical fractures and fracture non-unions or delayed unions. At the same time, there are preclinical data indicating that the osteoinductive action of teriparatide may contribute to the treatment of diseases such as osteogenesis imperfecta and osteonecrosis of the jaw. The possible induction of chondrogenesis may lead to the use of teriparatide for the treatment of osteoarthritis. Affinity with PTH has led to the use of teriparatide for the treatment of endocrine diseases such as hypoparathyroidism [6-8].

III. Off-label use of teriparatide in spine

A. Use of Teriparatide in Healing of Spinal Fractures

Rubery and Bukata investigated type III odontoid fractures in women with osteoporosis. These fractures are normally healed after immobilization for 8-12 weeks. The first case was a 91-year-old woman with persistent odontoid pseudarthrosis, depicted in CT, three months after the fracture. After seven months of daily administration of 20 μg teriparatide, the fracture was completely healed. The second case was an 84-year-old diabetic patient with odontoid non-union 15 weeks after the fracture. After 10 weeks of treatment with teriparatide (20 μg daily), fracture healing was seen in CT. Finally, an 82-year-old patient with multiple comorbidities had an odontoid non-union 16 weeks after the fracture; 20 μg teriparatide was administered daily and fracture union was observed four months after initiation of treatment [9].

B. Use of Teriparatide in Spinal Fusion Surgery

The aim of spinal fusion surgery is to achieve solid arthrodesis. The probability of fusion is maximized by the use of hardware during the first postoperative year, aiming to provide solid fixation, avoiding micro-motion. In osteoporotic patients, early hardware loosening is considered to decrease fusion rates. It is still debatable whether uninstrumented fusion rates are lower in comparison to those of instrumented fusion [10]. As a consequence, osteoporosis may compromise the results of spinal fusion by increasing the risk of early hardware loosening leading to non-union. In the osteoporotic spine, the optimization of bone-hardware interface is important as screw pullout is the most common cause of implant loosening [11].

The use of autografts is the gold standard for achieving spinal fusion. However, its use contains limitations because of the available amount of bone and potential donor-site morbidity. The aforementioned drawbacks and the increased rate of non-union have prompted surgeons to seek alternative means for the enhancement of bone formation. In order to accelerate spinal fusion, suppression of excessive osteoclastic resorption and promotion of bone formation of graft surfaces is of vital importance [12]. In the appropriately selected patients aged >70 years old, overall fusion rates are similar to those of younger patients [13]. Teriparatide may play an important role in increasing the success of spinal fusion surgery in osteoporosis, including the > 70-year-old age group.

Animal studies: Intermittent administration of teriparatide has accelerated spinal fusion in preclinical studies. Animal studies investigating fusion rates at four to six weeks have shown that teriparatide enhances fusion independent of its effects on the bone-screw interface. A study by Lawrence et al., in 2006, evaluated single-level, intertransverse process spinal fusion using iliac crest bone in 56 rats. A trend toward greater fusion rate was revealed in the teriparatide group (52%) compared to controls (37%), along with an increase in serum osteocalcin levels [14]. In another animal posterolateral fusion autograft study, daily subcutaneous administration of teriparatide in rats (40 μg/kg per day) increased the fusion rate in the experimental group (57%) in comparison to 14% in the control group, based on observations in 3D micro-CT images. Teriparatide resulted in a larger and denser fusion mass, along with an upregulation in gene expression of both osteoblast and osteoclast-related genes, and an increase of serum osteocalcin and CTX levels, histologically calculated mineral apposition rate, mineralized surface, and osteoclast surface. Authors have concluded that teriparatide accelerated and enhanced the healing process of bone grafts [15]. Morimoto et al. examined the effect of teriparatide administration combined with BMP transplantation in 48 rats that underwent posterolateral lumbar spinal arthrodesis. Authors have concluded that intermittent administration of teriparatide significantly increased fusion rates in animals treated with a low dose of rhBMP, improving the quality of newly formed bone and leading to efficient bone regeneration [16].

A randomized, controlled study by Qiu et al. assessed with micro-CT daily subcutaneous teriparatide administration (30 μg/kg per day) and spinal fusion in 36 rats with ovariectomy-induced osteoporosis that underwent bilateral posterolateral L4-L5 spinal fusion using iliac bone grafts. In comparison with the control group (56%), in the teriparatide group (89%), fusion rates and radiographic fusion scores were significantly higher, along with fusion bone volume, cortical thickness and serum NTX and osteocalcin (p < 0.05) [17]. Another study, by Sugiura et al., examined 18 rats with glucocorticoid-induced osteoporosis that underwent posterolateral spinal fusion (L4-L5) with iliac crest graft. Postoperatively, they received saline or teriparatide for six weeks. In the teriparatide group, fusion rate was higher (89%) compared with the control group (56%), along with the bone volume at the fusion mass [18]. The dose-dependent effect of teriparatide on lumbar intertransverse spinal fusion in rats was evaluated by Ming et al. Rats were randomly assigned to saline (group 1), 4 μg/kg/day teriparatide (group 2), and 23 μg/kg/day teriparatide (group 3) for four weeks. Administration of teriparatide in group 3 demonstrated anabolic skeletal effects and significantly promoted spinal fusion rate in rats, whereas administration of teriparatide in group 2 had also anabolic effects but did not significantly increase spinal fusion rate. Conclusively, teriparatide has a dose-dependent effect in enhancing spinal fusion [19]. 

In a study by O’Loughlin et al., 44 rabbits underwent bilateral posterolateral spinal fusion and were equally randomized to receive either daily teriparatide subcutaneous injections or a saline injection. Fusion was demonstrated by micro-CT in 81% of the teriparatide group and 30% of the control group (p<0.001). Teriparatide improved the quantity of the fusion bone produced and the histologic determinants compared to control [20]. In another animal study, 51 rabbits underwent a posterolateral L5-L6 intertransverse arthrodesis with the use of iliac crest bone graft and postoperatively were randomly assigned to a daily subcutaneous injection of placebo, teriparatide (10 μg/kg per day), or calcitonin for eight weeks. Histologic fusion rates for teriparatide averaged 86.7%, and were significantly higher than fusion rates of 50% in the control group (p = 0.033). Radiographically, in comparison to calcitonin, there was a strong trend towards teriparatide for higher fusion rates (86.7% vs 56.3%, p = 0.07) [21]. The third study with the use of a rabbit model was published by Lina et al., involving six study groups with the addition of rhBMP-2 use with teriparatide (10 μg/kg per day). When high doses of rhBMP - 2 in addition to teriparatide were administered, the fusion rates were 100%. Despite the increased fusion mass volume, teriparatide did not improve biomechanical stiffness in comparison to isolated use of autografts [22].

Conclusively, in multiple animal studies, teriparatide has increased spinal fusion rates and improved bone mineral density in comparison to controls, suggesting promising results in fusion success in patients with osteoporosis. No preclinical study has shown impairment of spinal fusion with teriparatide treatment. The addition of high dose rhBMP-2 and bisphosphonates has given promising results. 

Clinical studies: Treatment of osteoporotic women with teriparatide increased the spinal fusion rate following posterior lumbar fusion or posterior lumbar interbody fusion. Two recent studies have shown that teriparatide administration may reduce hardware pullout following spinal instrumentation. The incidence of pedicle screw loosening has been reported 0.6-27% in all patient groups. In a study by Ohtori et al., the effectiveness of teriparatide and bisphosphonates was compared in reducing pedicle screw loosening after instrumented posterolateral lumbar fusion with autograft bone. Sixty-two postmenopausal osteoporotic women suffering from degenerative spondylolisthesis were assigned into 3 treatment groups receiving daily subcutaneous teriparatide (n = 20), oral risedronate (n = 20), or no treatment (n = 22) for osteoporosis. Teriparatide and risedronate were administered two months before surgery and 10 months after surgery. One year after fusion, the rate of pedicle screw loosening was 7-13% in the teriparatide group, 13-26% in the risedronate group and 15-25% in the control group (p < 0.05). Teriparatide has increased the quality of lumbar spine pedicle bone [23].

Another study by Inoue et al. evaluated the efficacy of preoperative teriparatide administration in pedicle screws loosening, by measuring their insertional torque during fusion surgery. Twenty-nine postmenopausal women with osteoporosis underwent spinal fusion surgery. One group (n = 13) received subcutaneous teriparatide before surgery and a second group (n = 16) received no anti-osteoporotic treatment for at least one month. In the teriparatide group, the mean insertional torque was significantly higher in comparison to the control group (p < 0.01). Preoperative teriparatide administration was effective in increasing the insertional torque of pedicle screws during spinal fusion in osteoporotic patients [24].

In a prospective trial, Ohtori et al. evaluated the effect of teriparatide on bone union after instrumented lumbar posterolateral fusion with local autograft. Fifty-seven postmenopausal women suffering from osteoporosis diagnosed with degenerative spondylolisthesis were separated into 2 groups. Two months preoperatively and 10 months postoperatively, the first group (n = 29) received daily subcutaneous injection of teriparatide (20 μg/day), while the second group (n = 28) received weekly oral risedronate (17.5 mg/week). The rate of bone fusion was 82% in the first group, and 68% in the risedronate group. The average duration of bone fusion was eight months in the first group, and 10 months in the risedronate group. The authors concluded that daily subcutaneous teriparatide injection in postmenopausal women with osteoporosis after posterolateral fusion surgery produced better bone union than oral risedronate and that teriparatide treatment reduced the healing time for spinal fusion [25].

Ebata et al. conducted a multicenter, prospective, randomized study aiming to assess the role of weekly administration of teriparatide on patient outcome after posterior transforaminal lumbar interbody fusion. Sixty-six female osteoporotic patients who were >50 years of age, with lumbar degenerative disease were randomly allocated to receive teriparatide subcutaneously for six months after fusion or no teriparatide (control group). At four months after surgery, bone fusion was significantly higher in the teriparatide group. Teriparatide enhanced bone formation at the site of fusion and decreased bone resorption, within the early postoperative period [26].

In a recent study by Cho et al., 47 patients with osteoporosis underwent posterior lumbar interbody fusion with pedicle screw fixation for degenerative lumbar stenosis and instability. Participants of the study were separated into two groups. In group 1 (n = 23), 20 μg / day teriparatide was injected subcutaneously for three-month cycles alternating with three-month periods of oral sodium alendronate for 12 months. In group 2 (n = 24), 91 mg/week of alendronate was administered per os for ≥1 year. Clinical scores along with fusion rate, bony fusion duration, and bone mineral density changes were evaluated, by X-rays, CT and BMD. In the teriparatide group, the fusion was earlier than the alendronate group (6.0 ± 4.8 months vs 10.4 ± 7.2 months), and the bone fusion rate was higher at six months, without any difference at 12 and 24 months postoperatively. There was no significant difference in the clinical outcome while teriparatide seemed to increase BMD scores [27].

In an attempt to assess the combined influence of teriparatide and denosumab on spinal fusion after posterior lumbar interbody fusion, Ide et al. conducted a study in 16 patients with osteoporosis and lumbar stenosis who were randomly separated into two treatment groups after undergoing posterior lumbar interbody fusion with local bone grafts. Group 1 (n = 8) received teriparatide 20 μg / day, from one month preoperatively to one year postoperatively) and group 2 (n = 8) received 20 μg / day of teriparatide from one month preoperatively to one year postoperatively with an addition of 60 mg denosumab, administered at two and eight months after surgery. In group 1, alkaline phosphatase (ALP) increased more than in group 2 at three months postoperatively (p < 0.05). Bone mineral density in the femoral neck increased more in group 2 than in group 1 at one year postoperatively. In group 2, fusion rates were higher than in group 1 at six months postoperatively. Authors concluded that combination treatment with teriparatide and denosumab resulted in an increase of bone mineral density in comparison to teriparatide alone, and accelerated spinal fusion after posterior lumbar interbody fusion [28].

A meta-analysis published in 2018, including nine studies, evaluated the efficacy of teriparatide in thoracolumbar spinal fusion. The use of teriparatide was associated with higher fusion rates and decreased incidence of screw loosening than bisphosphonates. However, no statistically significant difference was noted between teriparatide and the risk of screw loosening [28]. Apart from the positive effects in the rate of spinal fusion, a recent retrospective study has shown that preoperative and postoperative administration of teriparatide may significantly reduce the incidence of subsequent vertebral fractures after instrumented fusion surgery for osteoporotic vertebral fractures [29]. Along with the animal studies, the majority of clinical studies show favourable results for the efficacy of teriparatide use in spine fusion rates. Notably, most of the published clinical data derive from studies in osteoporotic women leaving men as an understudied group of patients. 

## Conclusions

Teriparatide is the only FDA-approved anti-osteoporotic agent with an anabolic action. Its unique properties have led to the use of teriparatide as a means to augment spinal fusions, in animal and clinical studies. Intermittent administration of teriparatide shows potential as an adjunctive intervention for women with osteoporosis undergoing fusion surgery with the optional use of autologous bone grafts. The identification of the optimal teriparatide dosage and administration regimen for clinical application remains to be clarified. Future studies are needed to determine the exact timing of teriparatide administration to achieve maximum results, and the duration of administration required to optimize efficacy. Moreover, teriparatide administration on fusion rates should be tested in osteoporotic men. For a better understanding of the effects of teriparatide administration on instrumented spinal fusion, a study comparing teriparatide with standard anti-osteoporotic treatment is necessary.
